# Switching from allopurinol to febuxostat: efficacy and tolerability in hemodialysis patients

**DOI:** 10.1186/s40780-015-0028-1

**Published:** 2015-10-06

**Authors:** Satoru Mitsuboshi, Hitoshi Yamada, Kazuhiko Nagai, Hideo Okajima

**Affiliations:** Department of Pharmacy, Kaetsu Hospital, 1459-1 Higashikanazawa, Akiha-ku, Niigata-shi, Niigata 956-0814 Japan; Department of Internal Medicine, Kaetsu Hospital, 1459-1 Higashikanazawa, Akiha-ku, Niigata-shi, Niigata 956-0814 Japan

**Keywords:** Febuxostat, Allopurinol, Hemodialysis, Uric acid

## Abstract

**Background:**

Febuxostat is a novel xanthine oxidase inhibitor. However, few studies have examined the long-term efficacy and tolerability of febuxostat after switching from allopurinol in hemodialysis (HD) patients. Therefore, the present study evaluated the long-term efficacy and tolerability of febuxostat in HD patients after switching from allopurinol.

**Findings:**

We monitored the levels of hemoglobin, hematocrit, platelet count, blood urea nitrogen, serum creatinine, serum sodium, serum potassium, serum chloride, serum calcium, serum inorganic phosphorus, aspartate transaminase, alanine aminotransferase, alkaline phosphatase, lactate dehydrogenase, and total protein that were considered overall as a tolerability index, while the serum uric acid (UA) level was considered an index of efficacy. All values were measured at baseline and at 1, 6, 12, and 16 months after the switch to febuxostat therapy. All subjects switched from allopurinol (100 mg/day) to febuxostat (10 mg/day) in August 2013. Clinical laboratory data were collected at baseline in July 2013 until December 2014. Nine patients were included in the study analysis. Results showed that clinical laboratory data at baseline *versus* those at 16 months were not significantly different. Serum UA levels, which represented the efficacy index, were significantly different between the baseline level (6.8 ± 1.4) and those at 1, 6, 12, and 16 months (5.2 ± 1.1, 5.1 ± 1.1, 4.6 ± 0.9, and 5.4 ± 1.8 mg/dL, respectively; all *p* < 0.05).

**Conclusion:**

Switching from allopurinol to febuxostat in HD patients reduced serum UA levels, with no changes in other clinical laboratory data in the long term.

## Findings

### Background

Hyperuricemia is known to be associated with hypertension, vascular disease, chronic kidney disease (CKD), and cardiovascular disease (CVD) [1]. CVD is associated with a risk of morbidity and mortality in hemodialysis (HD) patients [2], and CKD patients receiving urate-lowering therapy are mainly treated with xanthine oxidase (XO) inhibitors [3].

Febuxostat is a novel XO inhibitor with urinary and fecal pathways of excretion (49.1 and 44.9 %, respectively) [4]. In a systematic review and meta-analysis study, febuxostat did not reduce the risk of gout flares compared with allopurinol, but the risk of adverse events was lower in febuxostat compared with allopurinol, and febuxostat was more likely than allopurinol to achieve serum uric acid (UA) levels of <6 mg/dL [5]. In another study, the incidence of adverse events was similar between allopurinol and febuxostat [6]. These studies suggest that allopurinol is a safe option in non-CKD patients. Oxypurinol, the major metabolite of allopurinol, is excreted *via* the kidney [7]. Some studies have demonstrated an increased risk of allopurinol-induced adverse reactions in CKD patients; however, the studies evaluating allopurinol use in CKD patients have shown inconsistent findings in relation to its safety and efficacy [8].

In HD patients, febuxostat therapy lowered serum UA levels significantly after 6 months [8]. In CKD patients, switching from allopurinol to febuxostat significantly decreased serum UA levels after 12 months [9]. However, to date, few studies have examined the long-term efficacy and tolerability of febuxostat when switching from allopurinol in HD patients. Side effects of febuxostat therapy for hyperuricemia in CKD patients have been reported [9, 10], but few studies have examined the pharmacokinetics of febuxostat in HD patients [11]. In HD patients, we hypothesized that efficacy and toxicity may be increased because serum concentrations of febuxostat might be higher than those in non-HD patients. Therefore, in this study we evaluated the long-term efficacy and tolerability of febuxostat in HD patients after switching from allopurinol.

### Methods

We conducted this 16-month prospective observational study of HD patients who had been receiving allopurinol at a dose of 100 mg/day for over 1 year. We excluded patients who received allopurinol at a dose of 50 or 150 mg/day, because few patients had been receiving allopurinol at these doses. We evaluated liver dysfunction in patients during the study period and also excluded patients with a history of active liver disease.

All the enrolled patients switched from allopurinol at 100 mg/day to febuxostat at 10 mg/day in August 2013. Clinical laboratory data were collected in the period from July 2013 to December 2014. Blood samples were obtained *via* vascular access before HD sessions. The levels of hemoglobin, hematocrit, platelet count, blood urea nitrogen, serum creatinine, serum sodium, serum potassium, serum chloride, serum calcium, serum inorganic phosphorus, aspartate transaminase, alanine aminotransferase, alkaline phosphatase, lactate dehydrogenase, and total protein were assessed and were considered overall as a tolerability index, while the serum UA level representing the efficacy index was measured at baseline (July 2013) and at 1, 6, 12, and 16 months after the switch to febuxostat therapy (August 2013).

This study was performed in accordance with the Declaration of Helsinki and was approved by the Ethics Committee of Kaetsu Hospital. Written informed consent was obtained from all patients before enrollment in the study.

All data are expressed as mean ± standard deviation. Statistical analysis was performed using the paired *t*-test, and one-way ANOVA followed by Hsu’s MCB test. Significance was set at *p* < 0.05. The software JMP 9 (SAS Institute Inc., Cary, NC) was used for all statistical analysis.

### Result

Twelve HD patients receiving allopurinol were considered as candidate subjects. Three patients were excluded from the study, including 2 who were receiving an allopurinol dose of 50 or 150 mg/day and 1 with a history of active liver disease. Therefore, we assessed 9 patients in this study. All 9 patients had oliguria or anuria, and there was no change in diuretic therapy. They did not receive any chronic steroid or non-steroidal anti-inflammatory drugs during the study period.

Patient profiles are shown in Table 1, and patient medications are shown in Table 2. Few changes to hemodialysis prescriptions were made in this study. One patient each had HD treatment time prolonged or shortened by 1 h, and the dialyzer membrane was changed for 2 patients. In regards to anti-hypertensive agents, angiotensin receptor blocker was added or withdrawn in 1 case each; calcium channel blocker was added in 1 case and withdrawn in 2; and alpha adrenergic blocker was withdrawn in 1 patient. In regards to angiotensin receptor blocker, patients had been administered azilsartan, candesartan, or olmesartan. HMG-CoA reductase inhibitor was added in 1 case. In regards to phosphate binders, lanthanum carbonate or sevelamer hydrochloride was added in 1 case each, and lanthanum carbonate was changed to sevelamer hydrochloride in 1 case. An iron agent was added in 1 case, withdrawn in 1, added and withdrawn in 6, and continued in 1 during the study period. As for potassium-binding resin, 1 patient each was administered calcium polystyrene sulfonate or sodium polystyrene sulfonate. The dose of each of the abovementioned medications was not changed during the study period. Eight patients were administered erythropoietin: the dose was increased in 4 patients, decreased in 1, and increased and decreased in 2 during the study period. All other medications were continued and their dosages were not changed during the study period.

**Table 1:** Patient profiles

*N*	9
Age (years)	62.3 ± 10.4
Sex (Female/Male)	1/8
Dry weight (kg)	59.7 ± 14.1
Hemodialysis duration (years)	12.4 ± 6.8
Etiology of renal disease (*n*)	
Glomerulonephritis	4
Polycystic kidney disease	3
Nephrosclerosis	1
Other	1
Cardiovascular disease (*n*)	
Hypertension	9
Hyperlipidemia	2
Chronic heart failure	3
Diabetes	1
Unstable angina pectoris	1
Cerebral infarction	1

Values are means ± standard deviation

**Table 2:** Patient medications

Medication	Start of the study	End of the study
Anti-hypertensive agents		
Angiotensin receptor blocker	4, (4)	4, (4)
Calcium channel blocker	5, (4)	6, (4)
Alpha adrenergic blocker	2, (2)	1, (1)
Beta blockers	2, (2)	2, (2)
Loop diuretic	1, (1)	1, (1)
HMG-CoA reductase inhibitors	1, (1)	2, (2)
Phosphate binders		
Calcium carbonate	7, (7)	7, (7)
Lanthanum carbonate	1, (1)	2, (2)
Sevelamer hydrochloride	5, (5)	6, (6)

number of medicine, (n)

Changes in clinical laboratory data (which represent the tolerability index) during the observation period are shown in Table 3. The tolerability index showed no statistical difference at baseline *versus* 16 months after the switch to febuxostat therapy.

**Table 3:** Changes in clinical laboratory data during the observation period

		Follow-up period (months)			
	Baseline	6	12	16	p*
Hemoglobin (g/dL)	10.9 ± 0.8	10.7 ± 0.9	10.3 ± 0.8	10.4 ± 0.7	0.23
Hematocrit (%)	32.6 ± 2.2	32.5 ± 2.8	30.9 ± 1.9	31.7 ± 2.6	0.47
Platelet count (×10^4^ cells/mm^3^)	13.6 ± 5.7	14.7 ± 6.4	14.1 ± 5.5	13.8 ± 5.4	0.71
Blood urea nitrogen (mg/dL)	65.9 ± 13.6	66.0 ± 11.3	57.5 ± 9.3	66.3 ± 12.3	0.94
Serum creatinine (mg/dL)	13.1 ± 2.5	12.8 ± 2.0	12.3 ± 1.9	12.4 ± 2.1	0.14
Serum sodium (mEq/L)	137.1 ± 1.8	138.1 ± 2.0	138.8 ± 1.4	138.2 ± 2.3	0.13
Serum potassium (mEq/L)	5.6 ± 0.5	5.2 ± 0.5	5.1 ± 0.5	5.7 ± 0.5	0.40
Serum chloride (mEq/L)	107.6 ± 2.1	106.8 ± 2.9	109.1 ± 2.2	108.4 ± 1.8	0.35
Serum calcium (mEq/L)	8.6 ± 0.3	8.5 ± 0.5	8.6 ± 0.4	8.6 ± 0.6	0.81
Serum inorganic phosphorus (mEq/L)	4.5 ± 0.6	4.9 ± 0.9	4.3 ± 0.8	5.0 ± 1.0	0.27
Aspartate transaminase (U/L)	10.2 ± 3.2	11.3 ± 4.4	9.2 ± 3.2	12.8 ± 5.4	0.21
Alanine aminotransferase (U/L)	10.4 ± 3.0	10.2 ± 3.3	9.8 ± 4.5	11.7 ± 4.7	0.54
Alkaline phosphatase (U/L)	196.6 ± 53.6	217.6 ± 82.6	198.0 ± 48.0	222.3 ± 67.1	0.09
Lactate dehydrogenase (U/L)	155.2 ± 27.1	157.6 ± 29.5	165.8 ± 23.3	165.3 ± 23.1	0.30
Total protein (g/dL)	5.9 ± 0.2	6.0 ± 0.4	5.9 ± 0.4	5.9 ± 0.3	0.63

Values are means ± standard deviation

*p < 0.05 between baseline and 16 months, paired *t*-test

**Fig. 1:**
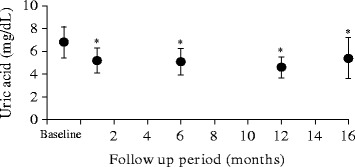
The effect of switching from allopurinol to febuxostat on serum UA levels

The effect of switching from allopurinol to febuxostat on serum UA levels is shown in Fig. 1. The serum UA level was significantly higher at baseline (6.8 ± 1.4) than at 1, 6, 12, and 16 months, respectively (5.2 ± 1.1, 5.1 ± 1.1, 4.6 ± 0.9, and 5.4 ± 1.8 mg/dL, respectively; all *p* < 0.05). Serum UA levels < 6 mg/dL were noted in 3 patients (33.3 %) at baseline and in 6 patients (66.7 %) at 16 months. Febuxostat dosage was reduced to 5 mg/day from 10 mg/day at 12 months in 1 patient because serum UA level before HD sessions had decreased to 4.4 mg/dL from 5.7 mg/dL. Febuxostat dosage was not changed in other patients during the study period. None of the patients withdrew from this study because of side effects or allergic reactions.

### Discussion

Our study revealed that switching from allopurinol to febuxostat in HD patients reduced serum UA levels, with no changes in other clinical laboratory data. As shown in Fig. 1, serum UA levels were 6.8 ± 1.4 and 5.4 ± 1.8 mg/dL at baseline and at 16 months after the start of the study, respectively, with levels < 6 mg/dL noted in 3 patients (33.3 %) and 6 patients (66.7 %) at these times, respectively. In the previous study by Akimoto et al. [8], febuxostat was shown to lower serum UA levels significantly from 1 month to 6 months after initiation of the treatment in HD patients, and serum UA levels < 6 mg/dL were achieved in 82.3 % of patients after 6 months of therapy. In the other study by Tsuruta et al. [9] in which CKD (non-HD) patients switched from allopurinol to febuxostat therapy, serum UA levels were 6.2 ± 0.9 mg/dL and 5.7 ± 1.2 mg/dL at 3 months before and 12 months after the switch, respectively; serum UA levels < 6 mg/dL were achieved in 45.1 and 68.6 % of patients at these times after the switch, respectively. Shoji et al. reported that serum urate concentrations < 6 mg/dl reduced the frequency of or prevented gout attacks [12]. The efficacy of febuxostat in our study was similar; febuxostat therapy may also reduce the risk of gout attacks in HD patients. Moreover, this efficacy was maintained until 16 months after the switch in therapy. In the present study, all patients began treatment with febuxostat at a dose of 10 mg/day, switching from an allopurinol dose of 100 mg/day. We considered the febuxostat dosage was an appropriate starting dose given that the majority of the HD patients in the study by Akimoto et al. [8] were treated with 10 mg of febuxostat and their serum UA levels decreased significantly from baseline values.

As shown in Table 3, clinical laboratory data (i.e., the tolerability index) showed no significant changes during the period from baseline to 16 months after the switch to febuxostat therapy. Similarly, in the study by Akimoto et al. [8], clinical laboratory data (representing the tolerability index) also showed no significant changes from baseline to 6 months after the start of the study. Febuxostat tolerability in our study was therefore similar, but was continued over 16 months. We consider that febuxostat is a safer therapy than allopurinol in CKD patients since some studies have demonstrated they have an increased risk of allopurinol-induced adverse reactions [3]. On the other hand, febuxostat therapy for hyperuricemia in CKD patients has been reported to cause acute neutropenia [10] and liver dysfunction [9]; therefore, it is important to monitor for these side effects in CKD patients receiving febuxostat therapy.

Losartan, an angiotensin receptor blocker, has been shown to increase urinary UA excretion and lower serum UA levels [13], although this effect has not been observed with other angiotensin receptor blockers. Patients were not administered losartan in the present study. Sevelamer hydrochloride, which is a phosphate binder, was found to be associated with a significant reduction in serum UA levels [14], and it was added or withdrawn in 1 case each in the present study. Therefore, we consider that angiotensin receptor blockers do not affect serum UA levels but that sevelamer hydrochloride may affect them. Iron agents and erythropoietin dosages were changed, but the levels of hemoglobin did not differ significantly during the study period.

Blood pressure was not examined in this study; however, anti-hypertensive agents were added in 2 cases and withdrawn in 4. Few studies have examined the effect of febuxostat therapy on blood pressure in HD patients [8]. Based on our results, febuxostat therapy may have little effect on blood pressure in HD patients.

Our study has certain limitations. These include the small sample size, lack of a control group, and few female patients. Also, urate-lowering therapy in HD patients has many potential confounders. In a previous study, higher uric acid concentrations were associated with a lower mortality among HD patients; higher uric acid concentration is a known marker of better nutritional status in this population [15]. Therefore, urate-lowering therapy in HD patients should be the subject of further investigation. In addition, the serum concentration of febuxostat in our HD patients was not assessed. To date, few studies have examined the pharmacokinetics of febuxostat in HD patients [11]. Febuxostat Tmax and Cmax were not affected by CLcr; however, AUC_24_ was significantly associated with CLcr. In HD patients, we consider that efficacy and toxicity may be increased because serum concentrations of febuxostat may be higher than those in non-HD patients. Therefore, when febuxostat is administered to them, it is important to consider that serum concentrations of febuxostat may be higher than those in non-HD patients.

Overall, our results indicate that switching from allopurinol to febuxostat in HD patients at a starting dose of 10 mg/day can reduce serum UA levels, with no changes in other clinical laboratory data over the long term.

## Abbreviations

CKD: chronic kidney disease
CVD: cardiovascular disease
HD: hemodialysis
XO: xanthine oxidase
UA: uric acid
